# Irritable Bowel Syndrome Is Positively Related to Metabolic Syndrome: A Population-Based Cross-Sectional Study

**DOI:** 10.1371/journal.pone.0112289

**Published:** 2014-11-10

**Authors:** Yinting Guo, Kaijun Niu, Haruki Momma, Yoritoshi Kobayashi, Masahiko Chujo, Atsushi Otomo, Shin Fukudo, Ryoichi Nagatomi

**Affiliations:** 1 Nutritional Epidemiology Institute and School of Public Health, Tianjin Medical University, Tianjin, China; 2 Department of Behavioral Medicine, Tohoku University Graduate School of Medicine, Sendai, Japan; 3 Division of Biomedical Engineering for Health & Welfare, Tohoku University Graduate School of Biomedical Engineering, Sendai, Japan; University Heart Center Freiburg, Germany

## Abstract

Irritable bowel syndrome is a common gastrointestinal disorder that may affect dietary pattern, food digestion, and nutrient absorption. The nutrition-related factors are closely related to metabolic syndrome, implying that irritable bowel syndrome may be a potential risk factor for metabolic syndrome. However, few epidemiological studies are available which are related to this potential link. The purpose of this study is to determine whether irritable bowel syndrome is related to metabolic syndrome among middle-aged people. We designed a cross-sectional study of 1,096 subjects to evaluate the relationship between irritable bowel syndrome and metabolic syndrome and its components. Diagnosis of irritable bowel syndrome was based on the Japanese version of the Rome III Questionnaire. Metabolic syndrome was defined according to the criteria of the American Heart Association scientific statements of 2009. Dietary consumption was assessed via a validated food frequency questionnaire. Principal-components analysis was used to derive 3 major dietary patterns: “Japanese”, “sweets-fruits”, and “Izakaya (Japanese Pub) “from 39 food groups. The prevalence of irritable bowel syndrome and metabolic syndrome were 19.4% and 14.6%, respectively. No significant relationship was found between the dietary pattern factor score tertiles and irritable bowel syndrome. After adjustment for potential confounders (including dietary pattern), the odds ratio (95% confidence interval) of having metabolic syndrome and elevated triglycerides for subjects with irritable bowel syndrome as compared with non-irritable bowel syndrome are 2.01(1.13–3.55) and 1.50(1.03–2.18), respectively. Irritable bowel syndrome is significantly related to metabolic syndrome and it components. This study is the first to show that irritable bowel syndrome was significantly related to a higher prevalence of metabolic syndrome and elevated triglycerides among an adult population. The findings suggest that the treatment of irritable bowel syndrome may be a potentially beneficial factor for the prevention of metabolic syndrome. Further study is needed to clarify this association.

## Introduction

Irritable bowel syndrome (IBS) is a common gastrointestinal disorder characterized by episodes of recurrent abdominal pain or discomfort related to disturbed bowel habits [1], [2]. The majority of subjects with IBS are conscious that diet may play a role in triggering these episodes and therefore may avoid certain foods and changes in their dietary pattern [3]–[8]. Furthermore, IBS disrupts the digestion of food, or directly interferes with nutrient absorption [9]–[11].

Metabolic syndrome (MS) is a well-recognized constellation of risk factors for cardiovascular disease (CVD) [12], which remains a major cause of mortality and morbidity worldwide [13]. Accumulated evidence suggests that dietary factors are the cornerstone for the prevention and treatment of MS [14]–[16]. Furthermore, with respect to dietary factors studies, researchers have usually focused predominantly on the effects of individual nutrients and sometimes foods, but rarely on dietary patterns. However, daily diets are composed of a wide variety of foods containing complex combinations of nutrients. The surveys that examine a single nutrient in foods, or a single food, may not adequately account for complicated interactions and cumulative effects on human health. Therefore, compared with a single nutrient in foods or a single food, a dietary pattern study may be a more important tool for evaluation of the effects of diet on health [17], [18].

Irritable bowel syndrome status may affect the dietary pattern, food digestion, and nutrient absorption, which are important factors for the prevention and treatment of MS and/or its components. Therefore, it is speculated that IBS may be a potential risk factor for MS. However, few epidemiological studies have assessed the relationship between IBS status and MS and its components in an adult population.

At present, we have designed a cross-sectional study to determine whether IBS is related to MS among middle-aged people.

## Materials and Methods

### Study Population

The current analysis uses data from a population-based longitudinal study designed to investigate the lifestyle risk factors of CVD among Japanese adults. The methods are described in detail elsewhere [19], [20].

There were 1,208 subjects who had received a health examination including blood examinations in 2011. Of these, 1,163 subjects agreed to participate and provided informed consent for their data to be analyzed. Subjects were excluded if they did not provide any dietary information (n = 21) or did not answer the Rome III Modular Questionnaire (n = 46). Owing to these exclusions, the final cross-sectional study population comprised 1,096 subjects (mean [standard deviation, SD] age: 46.2 [11.2] years; male, 77.5%). The Institutional Review Board of the Tohoku University Graduate School of Medicine approved the study protocol.

### Assessment of IBS

The Japanese version of the Rome III Questionnaire was used to screen for IBS [21]. All subjects were asked to complete self-reported ROME III diagnostic questionnaires. Screening for IBS requires that subjects have abdominal discomfort or pain lasting at least 3 days per month, not necessarily consecutive, during the previous 3 months which is associated with 2 or more of the following: relief by defecation; onset associated with a change in frequency of stool; onset associated with a change in form (appearance) of stool.

### Assessment of MS and Other

Waist circumference was measured at the umbilical level with participants standing and breathing normally. Blood pressure (BP) was measured twice from the upper left arm using a YAMASU605P automatic device (Kenzmedico, Saitama, Japan) after 5 min of rest in the seated position. The mean of these 2 measurements was taken as the BP value. Blood samples were collected in siliconized vacuum glass tubes containing sodium fluoride, for the analysis of fasting blood glucose (FBG), or containing no additives, for the analysis of lipids. Fasting blood glucose was measured by using enzymatic methods (Eerotec, Tokyo, Japan). The concentrations of triglycerides (TG), low-density lipoprotein cholesterol (LDL), and high-density lipoprotein cholesterol (HDL) were measured by enzymatic methods using appropriate kits (Sekisui Medical, Tokyo, Japan). Serum high-sensitive C-reactive protein (hsCRP) levels were determined using N-latex CRP-2 (Siemens Healthcare Japan, Tokyo, Japan). The measurement limit of hsCRP was 0.02 mg/L and an hsCRP value less than the measurement limit was considered to be 0.01 mg/L.

Metabolic syndrome was defined in accordance with the criteria of the American Heart Association scientific statements of 2009 [22]. Participants were considered to have MS when they presented three or more of the following components: 1) elevated waist circumference for Asian individuals (≥90 cm and ≥80 cm in male and female, respectively), 2) elevated TG (≥150 mg/dL), or drug treatment for elevated TG, 3) reduced HDL (<40 mg/dL in male; <50 mg/dL in female) or drug treatment for reduced HDL, 4) elevated blood pressure (SBP ≥130 mm Hg and/or DBP ≥85 mm Hg) or antihypertensive drug treatment, 5) elevated fasting glucose (≥100 mg/dL) or drug treatment of elevated glucose.

### Assessment of Dietary Intake

The subjects were instructed to complete a brief, self-administered diet history questionnaire (BDHQ) that included questions on 75 food items along with their specified serving sizes, described in terms of natural portions or standard weights and volume measures of the servings, commonly consumed by the study population. For each food item, the subjects indicated their mean frequency of consumption of the food over the past month in terms of the specified serving size by checking 1 of the 7 frequency categories, ranging from “almost never” to “2 or more times/day”. The mean daily consumption of nutrients was calculated using an *ad hoc* computer program developed to analyze the questionnaire. The Japanese food composition tables, 5^th^ edition, [23] and other [24] were used as the nutrient database. The reproducibility and validity of the BDHQ have already been described in detail elsewhere [25]. Foods from the BDHQ were categorized into 39 food subgroups, which were used to derive dietary patterns via principal-components analysis.

Factor analysis (principal-components analysis) was used to derive dietary patterns and to determine factor loadings for each of the 39 food subgroups (in g/d) [26]. Factors were rotated with varimax rotation to maintain uncorrelated factors and enhance interpretability [27]. A combined evaluation of the eigenvalues, scree plot test, and factor interpretability was used in determining the number of retained factors. The distinctive dietary patterns of the study population were well described by the 3 factors. Factors were named descriptively according to the food items showing high loading (absolute value) with respect to each dietary pattern as follows: “Japanese” dietary pattern (factor 1), “sweets-fruits” pattern (factor 2), and “Izakaya (Japanese Pub)” pattern (factor 3) (see **Table S1**). For each dietary pattern and each subject, we calculated a factor score by summing the consumption from each food item weighted by its factor loading as follows [27]: 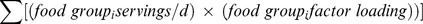
where *i* =  food groups 1–39. A higher factor score indicates greater conformity to the dietary pattern. Variables unrelated to a given dietary pattern are weighted close to zero. For further analyses, factor scores were categorized into 3 equal groups by using tertiles cutoffs.

### Assessment of Other Variables

Sociodemographic variables, including age, gender, and educational levels were also assessed. The educational level was assessed by determining the last grade level and was divided into 2 categories: <college or ≥college. Body mass index (BMI) was calculated as weight in kilograms divided by squared height in meters (kg/m2). History of physical illness and current medication were noted from “yes” or “no” responses to relevant questions. Information on smoking status, and drinking status were obtained from a questionnaire survey. Levels of daily physical activity (PA) were estimated using the International Physical Activity Questionnaire (IPAQ) (Japanese version) [28]. Total daily PA (metabolic equivalents [METs]×hours/week) were calculated as follows: (daily hours of walking × days per week with walking×3.3)+(daily hours of moderate-intensity activity×days per week with moderate-intensity activity×4.0)+(daily hours of vigorous activity×days per week with vigorous activity×8.0). The METs values were derived from the IPAQ validity and reliability study [28]. Physical activity was categorized into three groups: no PA, low PA (0<PA<23 METs × hours/week), and high PA (≥23 METs×hours/week) [29]. Depressive symptoms were assessed according to the Japanese version of the Self-Rating Depression Scale (SDS) [30]. An SDS score ≥45 was taken as the cutoff point indicating depressive symptoms [31].

### Statistical Analysis

All statistical analyses were performed using the Statistical Analysis System 9.1 edition for Windows (SAS Institute Inc., Cary, NC, USA). The age- and sex-adjusted variable differences according to IBS status were examined by analysis of covariance (ANCOVA) for continuous variables or by the multiple logistic regression analysis for variables of proportion. For main analysis, the MS or its components were used as a dependent variable and IBS status as independent variables. The odds ratio (OR) and 95% confidence interval (CI) of MS and its components compared with IBS status were calculated using multiple logistic regression analysis. We used age, sex, BMI, smoking and drinking status, educational level, PA levels, dietary patterns, total energy intake, depressive symptoms and mutual metabolic syndrome components as covariates for multiple adjustments. Model fit was evaluated using the Hosmer-Lemeshow goodness-of-fit statistic. For all models, the test was not significant (*P*≥0.31). Interactions between IBS status and confounders of MS or its components were tested by the addition of cross-product terms to the regression model. All tests were two-tailed and *P*<0.05 was defined as statistically significant.

## Results

Among 1,096 subjects who were available to be analyzed, 213 (19.4%) had self-reported IBS, and 160 (14.6%) had MS.

Age- and sex-adjusted characteristics of study subjects with and without IBS are presented in **Table 1**. Subjects with IBS were significantly younger than the non-IBS subjects (*P*<0.01) with a mean (95% CI) 43.4 (41.9–45.0) y compared to 46.1 (45.3–47.0) y. Subjects with IBS contained a lower proportion of males, and a higher proportion of ex-smokers and depressive symptoms (*P*<0.05 for all comparisons). Compared to subjects with IBS, the non-IBS subjects had lower total energy intake, and serum TG levels (*P*<0.05 for all comparisons).

**Table 1:** Age- and sex-adjusted characteristics of the subjects in relation to irritable bowel syndrome (n = 1,096)†

	Irritable bowel syndrome		*P* value‡
	No (n = 883)	Yes (n = 213)	
Age (y)	46.1 (45.3–47.0)§	43.4 (41.9–45.0)	<0.01
Sex (male, %)	79.2	70.4	0.02
BMI (kg/m2)	22.8 (22.5–23.0)	22.6 (22.1–23.1)	0.54
Smoking status (%)			
Current smoking	40.8	36.6	0.46
Ex-smoking	12.5	17.4	0.049
Never-smoking	46.6	46.0	0.53
Drinking status (%)			
Daily	28.3	25.4	0.77
Sometimes	48.5	50.2	0.93
Never-drinking	23.2	24.4	0.89
Educational level (≥ college, %)	33.8	28.2	0.14
PA (%)			
0 METs hours/week	25.4	23.5	0.50
0–23 METs hours/week	39.9	44.6	0.37
≥23 METs hours/week	34.8	31.9	0.76
Total energy intake (kcal/d)	1733.0 (1686.7–1779.4)	1836.6 (1754.4–1918.7)	0.02
“Japanese” dietary pattern			
The lowest tertile of factor score	32.7	35.7	0.85
The middle tertile of factor score	33.9	31.5	0.41
The highest tertile of factor score	33.4	32.9	0.48
“sweets-fruits” dietary pattern			
The lowest tertile of factor score	34.4	28.6	0.37
The middle tertile of factor score	33.6	32.4	0.44
The highest tertile of factor score	31.9	39.0	0.09
“Izakaya (Japanese Pub)” dietary pattern			
The lowest tertile of factor score	33.8	31.5	0.24
The middle tertile of factor score	33.9	31.5	0.55
The highest tertile of factor score	32.4	37.1	0.08
Depressive symptoms (SDS ≥45, %)	30.9	42.7	<0.01
Waist (cm)	80.2 (79.5–81.0)	80.6 (79.3–81.9)	0.61
SBP (mmHg)	122.1 (120.9–123.2)	121.6 (119.6–123.6)	0.67
DBP (mmHg)	75.7 (74.8–76.5)	75.1 (73.6–76.6)	0.49
Log translated TG (mg/dl)¶	86.5 (82.7–90.5)	98.4 (90.9–106.5)	<0.01
FBG (mg/dl)	96.7 (94.8–98.6)	96.4 (93.0–99.7)	0.85
HDL (mg/dl)	62.6 (61.5–63.7)	61.5 (59.6–63.4)	0.31
LDL (mg/dl)	114.1 (111.7–116.4)	114.5 (110.2–118.7)	0.87
Log translated hsCRP (mg/L)¶	0.32 (0.29–0.35)	0.30 (0.26–0.36)	0.64

† BMI, body mass index; PA, physical activity; METs, metabolic equivalents; SDS, Self-rating Depression Scale; SBP, systolic blood pressure; DBP, diastolic blood pressure; TG, triglyceride; FBG, fasting blood glucose; HDL, high-density lipoprotein-cholesterol; LDL, low-density lipoprotein; hsCRP, high-sensitivity C-reactive protein.

‡ Analysis of covariance or logistic regression analysis adjusted for age and sex where appropriate.

§ Adjusted least squares mean (95% confidence interval) (all such values).

¶ Adjusted geometric mean (95% confidence interval).

Because IBS may affect the dietary pattern [32], as an initial step, we evaluated the relationships between dietary patterns and IBS. Three major dietary patterns were identified by factor analysis (**Table S1**). Factor 1, identified as a traditional “Japanese” dietary pattern was characterized by a high consumption of vegetables, seaweeds, soybean products, fish, fruits, miso soup, and green tea. Factor 2 was typified by a greater consumption of cake, ice cream, fruits, bread, dairy products, mayonnaise and lower consumption of alcohol (named the “sweets-fruits” pattern). Factor 3 was typified by a greater consumption of noodles, Squid, octopus, lobster, shellfish, meat, fish, cola, alcohol, coffee, mayonnaise, chicken egg, and bread (named the “Izakaya (Japanese Pub)” pattern). These 3 patterns explained 32.1% of the variance in dietary consumption (18.6% for factor 1, 7.5% for factor 2, and 6.0% for factor 3). Increasing the number of patterns did not materially increase the total proportion of variance in dietary consumption explained by the model. Daily food and nutrient consumption are presented according to tertiles of dietary pattern factor score in **Table S2**. Compared to subjects with factor scores in the lowest tertile for the “Japanese” dietary pattern, those in the highest tertile had a higher consumption of total meats, total fish, seaweeds, total vegetables, soybean products, total fruits, dairy products, green tea, black or oolong tea, total energy intake, animal protein, vegetable protein, animal fat, vegetable fat, carbohydrate, total fiber, calcium, and eicosapentaenoic acid (EPA) + docosahexaenoic acid (DHA), and lower consumptions of cola (*P* for trend <0.05). Compared to those in the middle “sweets-fruits” pattern tertile, subjects in the highest tertile had significant higher consumptions of total fish, total seaweeds, total vegetables, total fruit, dairy products, green tea, cola, total energy intake, animal protein, vegetable protein, animal fat, vegetable fat, carbohydrate, total fiber, calcium, EPA+DHA, and a lower consumption of alcohol (*P*<0.05). Compared to those in the lowest “Izakaya (Japanese Pub)” pattern tertile, subjects in the highest tertile had a higher consumption of total meats, total fish, seaweeds, coffee, cola, total energy intake, animal protein, vegetable protein, animal fat, vegetable fat, carbohydrate, total fiber, calcium, EPA+DHA, alcohol, lower consumption of total fruits, and dairy products (*P* for trend <0.01). The age- and sex-adjusted relationships between tertiles of dietary pattern factor score and IBS status are indicated in **Table 1**. No significant relationships between the tertiles of each dietary pattern and IBS status were observed. These results were unchanged when we adjusted for multiple confounding factors (see Table 2 model 5) (*P*>0.15 for all comparisons).

**Table 2:** Adjusted odds ratios and 95% confidence interval for the relationship between MS and IBS (n = 1,096) †

	IBS vs non IBS				
	Model 1‡	Model 2§	Model 3¶	Model 4 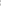	Model 5|
MS	1.85 (1.05–3.20)	1.94 (1.10–3.37)	2.01 (1.13–3.52)	2.01 (1.13–3.55)	-
MS components					
Waist circumference ≥90 cm for male or ≥80 cm for female	1.61 (0.89–2.92)	1.68 (0.91–3.06)	1.64 (0.88–3.03)	1.60 (0.86–2.97)	1.60 (0.85–2.96)
Triglycerides ≥150 mg/dL	1.52 (1.05–2.18)	1.53 (1.06–2.20)	1.52 (1.04–2.19)	1.51 (1.04–2.19)	1.50 (1.03–2.18)
HDL-cholesterol <40 mg/dL	1.04 (0.56–1.85)	0.99 (0.51–1.80)	1.00 (0.52–1.83)	1.04 (0.53–1.92)	1.00 (0.51–1.85)
SBP ≥130 mmHg or DBP ≥85 mmHg	1.04 (0.74–1.48)	1.03 (0.72–1.47)	1.05 (0.73–1.51)	1.06 (0.74–1.53)	1.04 (0.72–1.50)
High fasting glucose ≥100 mg/dL	1.27 (0.74–2.11)	1.24 (0.72–2.07)	1.26 (0.73–2.12)	1.25 (0.72–2.11)	1.19 (0.68–2.03)

† MS, metabolic syndrome; IBS, irritable bowel syndrome; HDL, high-density lipoprotein cholesterol; SBP, systolic blood pressure; DBP, diastolic blood pressure.

‡ Adjusted for age, sex and body mass index.

§ Additionally adjusted for smoking and drinking status, educational level, and physical activity.

¶ Additionally adjusted for dietary patterns, and total energy intake.

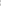
Additionally adjusted for depressive symptoms.

| Additionally adjusted for mutual metabolic syndrome components.

We next investigated whether IBS status is related to MS and its components. **Table 2** shows the adjusted relationships between IBS status and MS and its components. In the final multivariate models, the adjusted ORs (95% CI) of MS related to IBS group as compared with the non-IBS group is 2.01 (1.13–3.55). In MS components analysis, IBS status was only positively related to elevated TG in the final model (OR [95% CI]: 1.50 [1.03–2.18]). Although the difference was not statistically significant, the proportion of subjects with elevated waist circumference was higher in IBS group (OR [95% CI]: 1.60 [0.85–2.96]) in the final multivariate models. No significant relationships were observed between IBS status and other MS components in the final multivariate models. The tests for interactions between IBS status and other potential confounders in the final models were also not statistically significant (interaction P values>0.22).

## Discussion

In this cross-sectional study, we investigated the relationships between IBS and MS in an adult population. This study is the first to show that IBS is independently related to a higher prevalence of MS and elevated TG. Further, no significant relationships between IBS and dietary patterns were observed.

In this study, we adjusted for various potential confounders related to IBS and/or MS. First, we considered that age, sex (see Table 1), and body mass index [33] were potential confounders. Second, the effect of lifestyle factors, such as smoking [33] and drinking status [34], physical activity [35], and educational level [36], were adjusted. Moreover, IBS can affect dietary intake and thus affect the dietary pattern. Furthermore, dietary factors are also important for incidence of MS [35]. Accordingly, we made adjustments for total energy intake, and dietary pattern. Third, depressive symptoms are also closely related to IBS (see Table 1) and MS [37]. However, adjustments for these confounding factors did not change the significant positive relationship between IBS and MS. That is, the positive relationship between IBS and MS was independent of these factors.

It is hypothesized that IBS has a potentially adverse effect on MS and its components possibly due to effect on dietary pattern, food digestion, or nutrient absorption. However, no significant relationships between IBS and dietary patterns were observed. The result was similar to a previous study, suggesting that IBS is not related to dietary habits and/or nutritional intake [38]. Furthermore, although several studies have demonstrated self-reported food intolerances in most patients with IBS [3]–[8], two other studies have indicated that those with IBS appear to have adequate and balanced food and macronutrient intake, with no evidence of inadequate micronutrient intake [39], [40]. Therefore, we consider that while IBS symptoms affect the choice of certain specific food items, it has no essential impact on dietary pattern or the intake of nutrients. In the modern world, especially in developed countries, a variety of different foods are available in daily life. The availability of these choices may well make up for IBS symptoms-bringing harmful effects from certain food items. Further study is needed to confirm this hypothesis.

Despite underlying causes of pathophysiologic changes still not being completely understood, low grade mucosal inflammation, increased intestinal mucosal permeability, and abnormal intestinal motility are accepted mechanisms which alter gut function and generate symptoms of IBS [41]. The response of the gastrointestinal tract to ingestion of food is a complex and closely controlled process, which allows optimization of propulsion, digestion, absorption of nutrients, and removal of indigestible remnants. Therefore, it is believed that IBS is an important risk factor in the digestion of food and in nutrient absorption. Many studies have investigated the effects of IBS on the digestion of food or nutrient absorption [9]–[11]. These studies have consistently demonstrated that increased and discordant absorption of some nutrients, such as mannitol and sorbitol occurs in subjects with IBS compared to healthy controls [9]–[11]. In the present study, we found that IBS is mainly related to elevated TG, suggesting that IBS may affect the digestion and absorption of fats in the gastrointestinal tract. In fact, several studies have evaluated the relationships between IBS and the digestion and absorption of fats [8], [42]. Simren et al. have reported IBS to be related to increased colonic sensitivity and an altered viscerosomatic referral pattern after duodenal lipids infusion [42]. Another study also indicated that gastrointestinal symptoms were frequently reported after intake of fried and fatty foods in IBS patients [8]. Therefore, we consider the relationship between IBS and MS and its components to be possibly due to the disorder of food digestion and nutrient absorption, especially in the fat components. This study, which was designed to investigate the relationships between IBS and MS, is very limited and we therefore cannot determine an exact mechanism to explain our observations. Further studies are needed to make certain the causality and exact mechanisms of IBS in MS.

On the other hand, gut microbiota alterations could also be considered a potential link between IBS and MS and its components. The accumulated evidence has indicated that IBS is related to quantitative and qualitative changes in gut microbiota [43]. Because the gut microbiota is becoming known as a more and more important risk factor for the treatment and prevention of MS [44], [45], there is conjecture that IBS may be a potential risk factor for MS due to the effect of IBS on the quantitative and qualitative changes of gut microbiota. Further studies are needed to clarify this hypothesis.

A small-scale case-control study has shown that IBS was significantly related to a higher FBS and higher prevalence of prediabetes than in the control group [46]. In contrast, the present study did not find significant relationships between IBS and FBG or elevated FBG. Although the reason remains unclear, differences in age (mean age is 46.2 y in our study vs 33.0 y in their study), adjustment factors (all lifestyle factors were adjusted in their study) and population size may partly explain the discrepancy. Further study is needed to investigate this issue.

To the best of our knowledge, no previous study has examined the relationships between dietary pattern and IBS among the general population. The present study first investigated the relationships between dietary patterns and IBS in apparently healthy adults. The results suggest that dietary patterns were not related to the prevalence of IBS. Furthermore, the traditional Japanese diet is a well-known healthy diet pattern [26], [47]. Thus, we also evaluated whether the traditional Japanese dietary pattern was significantly related to a lower prevalence of IBS. The results indicated that no significant relationships between traditional Japanese dietary patterns and IBS were observed. Further study is needed to make certain of our observations.

This study had several limitations. First, the Rome III questionnaire is designed for the measurement and screening of IBS, not for making a clinical diagnosis. Therefore, a population study that uses a standardized comprehensive structured diagnostic interview should be undertaken to confirm the influence of IBS on MS. Second, because this study was a cross-sectional study, we could not conclude that IBS increases the occurrence of MS or that MS leads to episodes of IBS in adult populations. Therefore, a prospective study or trial should be undertaken to confirm the existence of a relationship between IBS, and MS and elevated TG.

In the present study, IBS was significantly related to a higher prevalence of MS and elevated TG among an adult population. The differences in dietary patterns are not likely to explain our findings. The findings suggest that the treatment of IBS may be a potentially beneficial factor for the development and prevention of MS. Further study is required to clarify this causality.

## Supporting Information

Table S1 Principal components analysis varimax-rotated 39 food groups factor loading scores (n = 1,096).
(DOC)

Table S2 Daily food and nutrient consumption of the participants according to the tertiles of dietary pattern factor score (n = 1,096).
(DOC)
