# Supplementary material for: Irritable Bowel Syndrome Is Positively Related to Metabolic Syndrome: A Population-Based Cross-Sectional Study
Source: PLoS One. 2014 Nov 10;9(11):e112289. doi: 10.1371/journal.pone.0112289 (PMC4226513; doi:10.1371/journal.pone.0112289)
Supplement: Table S1 — Principal components analysis varimax-rotated 39 food groups factor loading scores (n = 1,096). (DOC) [file pone.0112289.s001.doc]

| **Supplementary Table 1.** Principal components analysis varimax-rotated 39 food groups factor loading scores (n = 1,096). | | | |
| --- | --- | --- | --- |
|  | Dietary patterns | | |
| Food groups | "Japanese" | "sweets-fruits" | "Izakaya" |
| Cabbage | **0.75** | 0.08 | 0.09 |
| Carrot, pumpkin | **0.75** | 0.15 | -0.03 |
| Green-leaf vegetables | **0.73** | 0.14 | -0.10 |
| Japanese white radish (daikon), turnips | **0.72** | 0.11 | 0.01 |
| Other root vegetables (onion, burdock, and lotus root) | **0.70** | 0.15 | 0.05 |
| Mushrooms | **0.68** | 0.11 | 0.12 |
| Seaweeds | **0.68** | 0.11 | 0.12 |
| Potatoes | **0.66** | 0.10 | 0.10 |
| Vegetable salad (cabbage, lettuce) | **0.66** | 0.11 | -0.04 |
| Pickled vegetables (green-leaf vegetables) | **0.63** | 0.07 | 0.04 |
| Soybean (tofu) | **0.61** | 0.00 | 0.08 |
| Tomato | **0.57** | 0.18 | -0.06 |
| Fish (fish bone and all, dried fish, fatty fish, and less-fat fish) | **0.51** | 0.00 | **0.33** |
| Pickles (all kinds except for green-leaf vegetables) | **0.44** | 0.04 | 0.08 |
| Fermented soybeans | **0.40** | -0.01 | 0.12 |
| Mayonnaise | **0.40** | **0.24** | **0.21** |
| Meat and meat products (chicken, pork, beef, ham, sausage, bacon, and liver) | **0.38** | 0.01 | **0.41** |
| Chicken egg | **0.37** | 0.04 | **0.21** |
| Fruits (all kinds except for citrus fruits, persimmon, strawberry, and kiwi fruit) | **0.35** | **0.48** | -0.17 |
| Fruits (persimmon, strawberry, kiwi fruit) | **0.32** | **0.38** | -0.09 |
| Squid, octopus, lobster, shellfish | **0.32** | -0.06 | **0.49** |
| Dairy products (low-fat milk and yogurt, high-fat milk and yogurt) | **0.26** | **0.30** | -0.12 |
| Green tea | **0.25** | 0.15 | -0.03 |
| Miso soup | **0.24** | -0.05 | 0.18 |
| Fruits (citrus fruits) | **0.22** | **0.50** | 0.00 |
| Western-style cake, cookie, biscuit | 0.06 | **0.68** | 0.09 |
| Japanese cake | 0.13 | **0.62** | 0.14 |
| Rice cracker, rice cake, okonomiyaki | 0.17 | **0.58** | 0.14 |
| Ice cream | -0.04 | **0.53** | 0.14 |
| Bread | 0.00 | **0.44** | **0.20** |
| Alcohol consumption | 0.05 | **-0.43** | **0.27** |
| Noodles (udon, wheat noodles chilled on ice, thin wheat noodles, and Rahmen) | 0.01 | 0.12 | **0.70** |
| Noodles (buckwheat noodles) | 0.11 | 0.10 | **0.60** |
| Noodles (pasta) | 0.05 | 0.13 | **0.59** |
| Cola | -0.07 | 0.18 | **0.30** |
| Coffee | -0.02 | 0.03 | **0.22** |
| Rice | 0.17 | -0.07 | 0.18 |
| Black or oolong tea | 0.15 | 0.10 | 0.03 |
| 100% fruits or vegetables juice | 0.02 | 0.19 | 0.08 |
| Loadings with an absolute value more than 0.20 are shown in bold. | | | |
| Variance explained: 32.1%. | | | |
