# Supplementary material for: Irritable Bowel Syndrome Is Positively Related to Metabolic Syndrome: A Population-Based Cross-Sectional Study
Source: PLoS One. 2014 Nov 10;9(11):e112289. doi: 10.1371/journal.pone.0112289 (PMC4226513; doi:10.1371/journal.pone.0112289)
Supplement: Table S2 — Daily food and nutrient consumption of the participants according to the tertiles of dietary pattern factor score (n = 1,096). (DOC) [file pone.0112289.s002.doc]

| **Supplementary Table 2**. Daily food and nutrient consumption of the participants according to the tertiles of dietary pattern factor score (n = 1,096) † | | | | |
| --- | --- | --- | --- | --- |
|  | Tertiles of dietary pattern factor score | | | *P* for trend ‡ |
|  | Low | Middle | High |
| Food items (g/d) | (n = 365) | (n = 366) | (n = 365) |
| Total meats |  |  |  |  |
| "Japanese" | 50.0 (45.4, 54.6) § | 65.2 (60.6, 69.7) * | 85.2 (80.7, 89.8) *,** | < 0.0001 |
| "sweets-fruits" | 69.0 (64.3, 73.8) | 62.0 (57.2, 66.8) | 69.4 (64.6, 74.2) | 0.92 |
| "Izakaya (Japanese Pub)" | 51.6 (47.0, 56.2) | 63.3 (58.7, 67.9) * | 85.5 (81.0, 90.1) *,** | < 0.0001 |
| Total fish |  |  |  |  |
| "Japanese" | 29.1 (25.1, 33.1) | 51.1 (47.1, 55.1) * | 77.3 (73.3, 81.3) *,** | < 0.0001 |
| "sweets-fruits" | 57.2 (52.8, 61.7) | 44.8 (40.3, 49.2) * | 55.6 (51.1, 60.0) ** | 0.60 |
| "Izakaya (Japanese Pub)" | 40.4 (36.0, 44.7) | 49.7 (45.3, 54.0) * | 67.5 (63.2, 71.9) *,** | < 0.0001 |
| Seaweeds |  |  |  |  |
| "Japanese" | 4.6 (3.7, 5.6) | 10.0 (9.1, 10.9) * | 18.9 (18.0, 19.8) *,** | < 0.0001 |
| "sweets-fruits" | 10.9 (9.8, 12.0) | 9.5 (8.4, 10.6) | 13.2 (12.1, 14.3) *,** | < 0.01 |
| "Izakaya (Japanese Pub)" | 10.7 (9.6, 11.9) | 9.5 (8.4, 10.6) | 13.2 (12.1, 14.4) *,** | < 0.01 |
| Total vegetables |  |  |  |  |
| "Japanese" | 90.2 (80.8, 99.5) | 179.5 (170.2, 188.8) * | 340.9 (331.5, 350.2) *,** | < 0.0001 |
| "sweets-fruits" | 192.1 (178.1, 206.1) | 185.9 (171.9, 199.9) | 232.5 (218.5, 246.5) *,** | < 0.0001 |
| "Izakaya (Japanese Pub)" | 218.8 (204.8, 232.9) | 178.9 (164.9, 192.9) * | 212.8 (198.8, 226.9) ** | 0.56 |
| Soybean |  |  |  |  |
| "Japanese" | 47.2 (41.3, 53.0) | 84.1 (78.3, 90.0) * | 135.3 (129.4, 141.1) *,** | < 0.0001 |
| "sweets-fruits" | 92.9 (86, 99.8) | 84.0 (77.1, 90.9) | 89.7 (82.8, 96.6) | 0.52 |
| "Izakaya (Japanese Pub)" | 89.1 (82.2, 96.0) | 79.3 (72.4, 86.2) | 98.2 (91.3, 105.1) ** | 0.70 |
| Total fruits |  |  |  |  |
| "Japanese" | 22.2 (17.8, 26.7) | 36.0 (31.5, 40.4) * | 63.7 (59.3, 68.2) *,** | < 0.0001 |
| "sweets-fruits" | 19.1 (14.8, 23.3) | 32.7 (28.5, 36.9) * | 70.1 (65.9, 74.4) *,** | < 0.0001 |
| "Izakaya (Japanese Pub)" | 50.1 (45.3, 54.8) | 32.3 (27.6, 37.0) * | 39.5 (34.8, 44.2) * | < 0.01 |
| Dairy products |  |  |  |  |
| "Japanese" | 63.3 (52.9, 73.7) | 84.6 (74.3, 95.0) * | 123.5 (113.1, 133.8) *,** | < 0.0001 |
| "sweets-fruits" | 63.1 (52.8, 73.5) | 82.1 (71.8, 92.5) * | 126.1 (115.8, 136.5) *,** | < 0.0001 |
| "Izakaya (Japanese Pub)" | 106.7 (96.1, 117.3) | 89.2 (78.6, 99.8) | 75.5 (64.9, 86.1) * | < 0.0001 |
| Green tea |  |  |  |  |
| "Japanese" | 153.4 (133.0, 173.8) | 180.0 (159.6, 200.3) | 258.9 (238.5, 279.3) *,** | < 0.0001 |
| "sweets-fruits" | 172.3 (151.6, 192.9) | 180.5 (159.9, 201.2) | 239.4 (218.8, 260.1) *,** | < 0.0001 |
| "Izakaya (Japanese Pub)" | 219.7 (198.9, 240.5) | 171.8 (151.1, 192.6) * | 200.7 (179.9, 221.5) | 0.20 |
| Black or oolong tea |  |  |  |  |
| "Japanese" | 69.7 (55.1, 84.2) | 75.4 (60.9, 90.0) | 109.9 (95.3, 124.4) *,** | 0.0001 |
| "sweets-fruits" | 71.2 (56.6, 85.9) | 85.4 (70.8, 100.0) | 98.3 (83.7, 113.0) * | 0.01 |
| "Izakaya (Japanese Pub)" | 79.9 (65.2, 94.6) | 84.8 (70.1, 99.4) | 90.3 (75.6, 104.9) | 0.33 |
| Coffee |  |  |  |  |
| "Japanese" | 234.5 (214.6, 254.4) | 216.6 (196.7, 236.4) | 223.6 (203.7, 243.5) | 0.45 |
| "sweets-fruits" | 206.6 (186.8, 226.4) | 247.8 (228.0, 267.6) * | 220.2 (200.4, 240.0) | 0.34 |
| "Izakaya (Japanese Pub)" | 156.9 (137.6, 176.1) | 251.8 (232.5, 271.0) * | 265.9 (246.6, 285.2) * | < 0.0001 |
| Cola |  |  |  |  |
| "Japanese" | 124.1 (107.7, 140.5) | 110.1 (93.8, 126.5) | 100.4 (84.1, 116.8) | 0.045 |
| "sweets-fruits" | 77.6 (61.5, 93.8) | 105.0 (88.9, 121.1) * | 152.1 (136.0, 168.2) ** | < 0.0001 |
| "Izakaya (Japanese Pub)" | 52.0 (36.4, 67.6) | 109.8 (94.3, 125.4) * | 172.9 (157.3, 188.5) *,** | < 0.0001 |
| Nutrient items (g/d) |  |  |  |  |
| Total energy intake (kcal/d) |  |  |  |  |
| "Japanese" | 1548.0 (1490.3, 1605.6) | 1862.1 (1804.5, 1919.6) * | 2220.8 (2163.1, 2278.4) *,** | < 0.0001 |
| "sweets-fruits" | 1852.9 (1790.9, 1915.0) | 1693.5 (1631.6, 1755.4) * | 2084.8 (2022.8, 2146.9) *,** | < 0.0001 |
| "Izakaya (Japanese Pub)" | 1495.8 (1442.6, 1549.0) | 1794.8 (1741.7, 1847.9) * | 2340.4 (2287.2, 2393.5) *,** | < 0.0001 |
| Animal protein |  |  |  |  |
| "Japanese" | 23.9 (22.1, 25.6) | 35.0 (33.3, 36.8) * | 48.8 (47.1, 50.6) *,** | < 0.0001 |
| "sweets-fruits" | 36.3 (34.3, 38.3) | 31.9 (29.8, 33.9) * | 39.5 (37.5, 41.6) ** | 0.03 |
| "Izakaya (Japanese Pub)" | 28.9 (27.0, 30.8) | 34.2 (32.3, 36.1) * | 44.6 (42.7, 46.5) *,** | < 0.0001 |
| Vegetable protein |  |  |  |  |
| "Japanese" | 22.9 (22.0, 23.8) | 28.4 (27.5, 29.3) * | 35.8 (34.9, 36.7) *,** | < 0.0001 |
| "sweets-fruits" | 26.9 (25.9, 28.0) | 26.8 (25.8, 27.8) | 33.4 (32.4, 34.4) *,** | < 0.0001 |
| "Izakaya (Japanese Pub)" | 24.5 (23.6, 25.4) | 26.7 (25.8, 27.7) * | 35.9 (35.0, 36.9) *,** | < 0.0001 |
| Animal fat |  |  |  |  |
| "Japanese" | 17.2 (16.1, 18.2) | 22.7 (21.6, 23.8) * | 29.9 (28.8, 31.0) *,** | < 0.0001 |
| "sweets-fruits" | 21.1 (19.9, 22.2) | 20.9 (19.8, 22.1) | 27.8 (26.6, 28.9) *,** | < 0.0001 |
| "Izakaya (Japanese Pub)" | 19.3 (18.1, 20.4) | 22.5 (21.4, 23.7) * | 28.0 (26.8, 29.1) *,** | < 0.0001 |
| Vegetable fat |  |  |  |  |
| "Japanese" | 20.6 (19.6, 21.5) | 24.9 (24.0, 25.9) * | 31.0 (30.1, 32.0) *,** | < 0.0001 |
| "sweets-fruits" | 21.6 (20.6, 22.5) | 23.9 (22.9, 24.8) * | 31.1 (30.1, 32.1) *,** | < 0.0001 |
| "Izakaya (Japanese Pub)" | 21.4 (20.5, 22.4) | 23.7 (22.7, 24.6) * | 31.4 (30.5, 32.4) *,** | < 0.0001 |
| Carbohydrate |  |  |  |  |
| "Japanese" | 214.6 (205.6, 223.6) | 245.8 (236.9, 254.8) * | 285.5 (276.5, 294.5) *,** | < 0.0001 |
| "sweets-fruits" | 225.3 (216.3, 234.2) | 230.4 (221.5, 239.4) | 290.3 (281.4, 299.3) *,** | < 0.0001 |
| "Izakaya (Japanese Pub)" | 202.7 (194.4, 211.1) | 236.5 (228.2, 244.9) * | 306.7 (298.4, 315.1) *,** | < 0.0001 |
| Total fiber |  |  |  |  |
| "Japanese" | 6.9 (6.5, 7.2) | 10.2 (9.8, 10.5) * | 15.6 (15.2, 15.9) *,** | < 0.0001 |
| "sweets-fruits" | 9.9 (9.4, 10.4) | 9.9 (9.4, 10.3) | 12.9 (12.4, 13.4) *,** | < 0.0001 |
| "Izakaya (Japanese Pub)" | 10.1 (9.6, 10.6) | 9.6 (9.1, 10.1) | 12.9 (12.4, 13.3) *,** | < 0.0001 |
| Calcium (mg/d) |  |  |  |  |
| "Japanese" | 305.8 (286.6, 325.0) | 445.0 (425.8, 464.2) * | 662.8 (643.6, 682.0) *,** | < 0.0001 |
| "sweets-fruits" | 414.1 (391.0, 437.2) | 419.0 (395.9, 442.0) | 580.6 (557.5, 603.7) *,** | < 0.0001 |
| "Izakaya (Japanese Pub)" | 453.5 (429.3, 477.7) | 442.2 (418.0, 466.3) | 517.9 (493.7, 542.1) *,** | < 0.001 |
| EPA+DHA (mg/d) |  |  |  |  |
| "Japanese" | 429.1 (377.0, 481.3) | 722.1 (670.0, 774.2) * | 1068.3 (1016.1, 1120.4) *,** | < 0.0001 |
| "sweets-fruits" | 787.8 (729.6, 845.9) | 633.6 (575.5, 691.7) * | 798.4 (740.3, 856.6) ** | 0.80 |
| "Izakaya (Japanese Pub)" | 563.8 (507.6, 620.0) | 698.8 (642.7, 754.9) * | 957.0 (900.8, 1013.3) *,** | < 0.0001 |
| Alcohol |  |  |  |  |
| "Japanese" | 19.1 (16.2, 22.0) | 23.7 (20.9, 26.6) | 23.3 (20.4, 26.1) | 0.04 |
| "sweets-fruits" | 39.9 (37.4, 42.5) | 15.3 (12.7, 17.8) * | 10.9 (8.3, 13.5) * | < 0.0001 |
| "Izakaya (Japanese Pub)" | 12.3 (9.6, 15.1) | 22.8 (20.0, 25.5) * | 31.0 (28.2, 33.7) *,** | < 0.0001 |
| † EPA, eicosapentaenoic acid; DHA, docosahexaenoic acid. | | | | |
| ‡ Analysis of variance. | | | | |
| § Mean (95% confidence interval) (all such values). | | | | |
| * Significantly different from the lowest pattern score tertile (Bonferroni correction): *P <*0.05. | | | | |
| ** Significantly different from middle pattern score tertile (Bonferroni correction): *P <*0.05. | | | | |
